# Concordant peripheral lipidome signatures in two large clinical studies of Alzheimer’s disease

**DOI:** 10.1038/s41467-020-19473-7

**Published:** 2020-11-10

**Authors:** Kevin Huynh, Wei Ling Florence Lim, Corey Giles, Kaushala S. Jayawardana, Agus Salim, Natalie A. Mellett, Adam Alexander T. Smith, Gavriel Olshansky, Brian G. Drew, Pratishtha Chatterjee, Ian Martins, Simon M. Laws, Ashley I. Bush, Christopher C. Rowe, Victor L. Villemagne, David Ames, Colin L. Masters, Matthias Arnold, Kwangsik Nho, Andrew J. Saykin, Rebecca Baillie, Xianlin Han, Rima Kaddurah-Daouk, Ralph N. Martins, Peter J. Meikle

**Affiliations:** 1 Baker Heart and Diabetes Institute, Melbourne, VIC Australia; 2 Monash University, Melbourne, VIC 3800 Australia; 3 School of Medical and Health Sciences, Edith Cowan University, Perth, WA Australia; 4 Cooperative research Centre (CRC) for Mental Health, Sydney, NSW Australia; 5 Department of Mathematics and Statistics, La Trobe University, Melbourne, VIC Australia; 6 Melbourne School of Global and Population Health, The University of Melbourne, Melbourne, VIC 3010 Australia; 7 School of Mathematics and Statistics, The University of Melbourne, Melbourne, VIC 3010 Australia; 8 Department of Biomedical Sciences, Macquarie University, Sydney, NSW Australia; 9 KaRa Institute of Neurological Disease, Sydney, NSW Australia; 10 Collaborative Genomics Group, School of Medical and Health Sciences, Edith Cowan University, Perth, WA Australia; 11 Curtin Health Innovation Research Institute, Curtin University, Perth, WA Australia; 12 The Florey Department of Neuroscience and Mental Health, The University of Melbourne, Melbourne, VIC Australia; 13 Department of Nuclear Medicine and Centre for PET, Austin Health, Heidelberg, VIC Australia; 14 Department of Medicine, Austin Health, The University of Melbourne, Heidelberg, VIC Australia; 15 National Ageing Research Institute, Parkville, VIC 3050 Australia; 16 Department of Psychiatry and Behavioral Sciences, Duke University, Durham, NC USA; 17 Institute of Computational Biology, Helmholtz Zentrum München, German Research Center for Environmental Health, Neuherberg, Germany; 18 Department of Radiology and Imaging Sciences, Indiana University School of Medicine, Indianapolis, IN USA; 19 Center for Computational Biology and Bioinformatics, Indiana University School of Medicine, Indianapolis, IN USA; 20 Indiana Alzheimer Disease Center, Indiana University School of Medicine, Indianapolis, IN USA; 21 Department of Medical and Molecular Genetics, Indiana University School of Medicine, Indianapolis, IN USA; 22 Rosa & Co LLC, San Carlos, CA USA; 23 Barshop Institute for Longevity and Aging Studies, University of Texas Health Science Center at San Antonio, San Antonio, TX USA; 24 Duke Institute of Brain Sciences, Duke University, Durham, NC USA; 25 Department of Medicine, Duke University, Durham, NC USA; 26 School of Psychiatry and Clinical Neurosciences, The University of Western Australia, Perth, WA Australia; 27 Australian Alzheimer’s Research Foundation, Nedlands, WA Australia

**Keywords:** Lipidomics, Lipids, Metabolomics, Neurodegeneration, Diagnostic markers

## Abstract

Changes to lipid metabolism are tightly associated with the onset and pathology of Alzheimer’s disease (AD). Lipids are complex molecules comprising many isomeric and isobaric species, necessitating detailed analysis to enable interpretation of biological significance. Our expanded targeted lipidomics platform (569 species across 32 classes) allows for detailed lipid separation and characterisation. In this study we examined peripheral samples of two cohorts (AIBL, *n* = 1112 and ADNI, *n* = 800). We are able to identify concordant peripheral signatures associated with prevalent AD arising from lipid pathways including; ether lipids, sphingolipids (notably GM_3_ gangliosides) and lipid classes previously associated with cardiometabolic disease (phosphatidylethanolamine and triglycerides). We subsequently identified similar lipid signatures in both cohorts with future disease. Lastly, we developed multivariate lipid models that improved classification and prediction. Our results provide a holistic view between the lipidome and AD using a comprehensive approach, providing targets for further mechanistic investigation.

## Abstract (web summary)

The onset and pathology of Alzheimer’s disease (AD) is associated with changes to lipid metabolism. Here, the authors analysed 569 lipids from 32 classes and subclasses in two independent patient cohorts to identify key lipid pathways to link the plasma lipidome with AD and the future onset of AD.

## Introduction

Alzheimer’s disease (AD) is a neurodegenerative disease characterised by progressive decline in cognitive function, usually presenting with memory loss. In the sporadic form of AD, symptoms usually begin to manifest after the age of 65, and with the ageing global population, the number of people with AD has been estimated to reach 81 million worldwide by 2040 (ref. ^1^). The failure of many AD clinical trials over recent years has led to the call for a paradigm shift in AD research. It is now recognised that additional underlying mechanisms are involved in the pathogenesis of AD. We seek to provide a deeper molecular understanding of metabolic pathways implicated in AD to identify key enzymes, transporters and signalling molecules that are most amenable for therapeutic targeting. As there are no appropriate sporadic mouse models of AD, human studies are essential for better understanding pathogenesis of AD. In particular, statistically powered studies are needed to detect the associations beneath the natural human biological variation.

Lipids are fundamental to every living system. These diverse and biologically important molecules comprise thousands of individual species, spanning multiple classes and subclasses. In plasma, the majority of lipids are small amphiphilic molecules (including cholesterol) that make up the circulating lipoprotein particles such as high- and low-density lipoprotein (HDL and LDL). With recent advances to mass spectrometry and high-performance liquid chromatography, it is now feasible to examine in detail the comprehensive plasma lipidome in a human population or clinical study^2^. Quantification and characterisation of these diverse lipid molecules form the foundation of the field known as lipidomics.

Evidence that lipids are involved in AD have been demonstrated via alterations observed in phospholipid^3–5^, plasmalogens^6^, ceramide^7^, ganglioside^8^ and sulfatide^7,9^ compositions in the brain. Several recent studies indicate that altered phospholipid metabolism associated with AD pathogenesis is also observed in the blood^5,10,11^, thus encouraging discovery studies for blood-based lipid markers. Furthermore, a recent large-scale genome-wide association meta-analysis has identified genes involved in lipid metabolism as key risk factors for AD^12^.

The plasma lipidome is complex and consists of many isomeric and isobaric species^13^, these are species that share similar or identical elemental composition but might be structurally different and display specific associations with biological outcomes. Existing lipidomic studies often employ techniques that either have limited coverage of the lipidome (e.g., focusing on one or two lipid classes) and/or provide poor resolution of lipid species^5,14–17^. Limited specificity of lipidomic platforms can result in the aggregation of multiple species in one signal, limiting interpretation and reproducibility. Comprehensive approaches using untargeted lipidomics can provide greater coverage of the lipidome^18,19^, but can sometimes result in ambiguous identifications. More recent studies into lipidomics and dementia with updated methodology have shown that more structural granularity can lead to improved interpretation of results^20^. We have recently expanded our lipidomic platform to better characterise isomeric lipid species, now measuring 569 lipids from 32 classes and subclasses. Our methodology focuses on lipid and lipid-like compounds utilising chromatographic separation. We have applied this methodology to two large independent studies: The Australian Imaging, Biomarkers and Lifestyle (AIBL) flagship study of ageing^21^ and the Alzheimer’s Disease Neuroimaging Initiative baseline (ADNI) cohort. Here we show the importance of capturing the comprehensive lipidome and highlight the necessity of obtaining molecular structural detail to identify key lipid pathways to link the plasma lipidome with AD and the future onset of AD.

## Results

### Lipidomic analysis of the AIBL and ADNI cohort

Between the two cohorts, a total of 5733 samples (including quality controls and blanks) on 1912 unique individuals were analysed. The characteristics of individuals in the cross-sectional and longitudinal analysis are shown in Table 1 and further breakdowns are provided in Supplementary Table 1.

**Table 1:** Study characteristics.

AIBL	Prevalent analysis (latest time points^a^)			Incident AD analysis (baseline samples)		
	Control	AD	*P* value	Non-converters	Converters	*P* value
*n*	696	268		714	68	
Age (years)^b^	75.27 (6.53)	81.40 (7.91)	2.96 × 10^−^^32^	70.30 (6.90)	77.00 (6.86)	5.83 × 10^−14^
Gender (% female)^c^	408 (58.6)	159 (59.3)	0.899	412 (57.7)	36 (52.9)	0.529
BMI (kg/m^2^)^b^	26.29 (4.35)	25.52 (3.71)	0.01	26.46 (4.19)	24.77 (3.64)	0.001
Cholesterol (mmol/l)^b^	5.26 (1.12)	5.39 (1.31)	0.106	5.49 (1.06)	5.50 (1.08)	0.886
HDL-C (mmol/l)^b^	1.58 (0.43)	1.50 (0.41)	0.008	1.67 (0.45)	1.68 (0.51)	0.853
Triglycerides (mmol/l)^b^	1.26 (0.63)	1.50 (0.78)	9.42 × 10^−7^	1.31 (0.61)	1.34 (0.55)	0.784
Site (%Melbourne)^c^	402 (57.8)	185 (69.0)	0.002	398 (55.7)	44 (64.7)	0.195
ApoE (no. of ε4 alleles)^c^			7.31 × 10^−33^			1.02 × 10^−10^
0	523 (75.1)	99 (36.9)		516 (72.3)	25 (36.8)	
1	163 (23.4)	131 (48.9)		177 (24.8)	33 (48.5)	
2	10 (1.4)	38 (14.2)		21 (2.9)	10 (14.7)	
Time to conversion/last follow-up (years)				6.15 (2.18)	3.06 (2.03)	3.31 × 10^−^^27^
Number of MCI individuals (%)				47 (6.5)	50 (73.5)	2.63 × 10^−^^56^

ADNI	Prevalent analysis (baseline samples)			Incident AD analysis (baseline samples)		
	Control	AD	*P* value	Non-converters	Converters	*P* value
*n*	210	178		397	166	
Age (years)^b^	75.78 (4.93)	75.21 (7.52)	0.364	75.28 (6.34)	75.09 (7.06)	0.754
Gender (%female)^a^	103 (49.0)	88 (49.4)	1	164 (41.3)	64 (38.6)	0.608
BMI (kg/m^2^)^b^	26.76 (4.34)	25.57 (3.97)	0.005	26.69 (4.20)	25.69 (4.03)	0.009
Cholesterol (mmol/l)^b^	4.72 (0.94)	4.77 (0.94)	0.611	4.75 (0.97)	4.84 (1.09)	0.331
HDL-C (mmol/l)^b^	1.38 (0.45)	1.37 (0.44)	0.838	1.36 (0.44)	1.43 (0.46)	0.08
Triglycerides (mmol/l)^b^	1.43 (0.81)	1.42 (0.68)	0.951	1.40 (0.84)	1.36 (0.65)	0.586
No. of non-fasting	19 (9.0)	13 (7.3)	0.662	34 (8.6)	14 (8.4)	1
ApoE (No. ε4 alleles)^c^			1.52 × 10^−^^15^			1.91 × 10^−10^
0	154 (73.3)	60 (33.7)		257 (64.7)	58 (34.9)	
1	51 (24.3)	85 (47.8)		118 (29.7)	83 (50.0)	
2	5 (2.4)	33 (18.5)		22 (5.5)	25 (15.1)	
Time to conversion/last follow-up (years)				2.76 (0.91)	1.67 (0.89)	4.19 × 10^−34^
Number of MCI individuals (%)				197 (49.6)	163 (98.2)	2.04 × 10^−27^

^a^Latest time point utilises the most recent/last available sample for each participant out of all samples acquired for lipidomics.

^b^Two-group ANOVA.

^b^Chi-square.

**Fig. 1:**
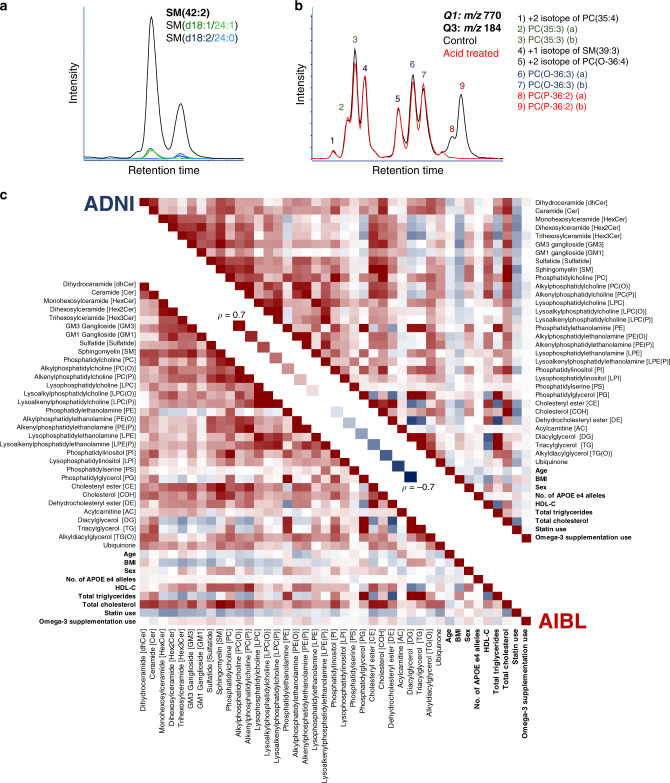
Characterisation of lipid isomeric species and the relationship of lipid classes and subclasses within the AIBL and ADNI cohorts. **a** Characterisation of sphingomyelin isomers. Black trace corresponds to the chromatogram seen under normal conditions. Additional experimental results in the green and blue traces used for identification, corresponding to SM(d18:1/24:1) and SM(d18:2/24:0) respectively. **b** Characterisation of glycerophospholipid isomers. Black trace corresponds to the chromatogram seen under normal conditions. Red trace is the same scan after sample acid hydrolysis. **c** Spearman correlation of total lipid classes, subclasses and commonly reported clinical measures (bolded) for the AIBL baseline and ADNI studies.

We developed our platform to better characterise the lipids in human plasma^22^. In total, we are able to examine 32 lipid classes and subclasses (conditions detailed in Supplementary Table 2) for both cohorts. In general, a similar correlation structure was observed within the AIBL and ADNI cohorts between lipid classes and many clinical measures (Fig. 1). Many commonly reported lipid species associated with AD, such as sphingolipids and ether lipids, are often reported with ambiguous annotations, where the reported species are the sum of several isomers. Here we report the detailed characterisation of these species.

Sphingolipids are structurally resolved through collision-induced dissociation (CID), where fragments correspond to the sphingoid base, with the exception of sphingomyelins. Dissociation of sphingomyelin species under normal conditions results in a product ions that yields only sum composition data, i.e. the sphingomyelin species, SM(42:2). To determine sphingomyelin structural composition, we repeated the mass spectrometry analysis on pooled plasma samples in the presence of lithium acetate as described previously^22^. The lithiated adduct of sphingomyelins produces product ions corresponding to the sphingoid base and *n*-acyl chain (Fig. 1a) allowing for structural identification. Alignment through chromatography highlights that our measurement of SM(42:2), for example, is chromatographically separated into SM(d18:1/24:1) and SM(d18:2/24:0) (Fig. 1a).

Similarly, this approach was repeated with the different glycerophospholipid classes to capture isomeric and isobaric structural details where they were chromatographically resolved. Examination of the transition *m/z* 770.6/184.1 corresponding to the phosphatidylcholine/alkylphosphatidylcholine/alkenylphosphatidylcholine species PC(35:3)/PC(O-36:3)/PC(P-36:2) results in nine distinct peaks (Fig. 1b). Here we report the complete separation of diacyl odd-numbered phosphatidylcholine species, the non-plasmalogen ether lipids PC(O) and the plasmalogen ether lipids, PC(P). Results were confirmed by exploiting the susceptibility of plasmalogens to acid hydrolysis (Fig. 1b). The correlation structure of all 32 classes and subclasses along with clinical variables is depicted in Fig. 1c.

### Concordance of associations between two studies with AD

**Fig. 2:**
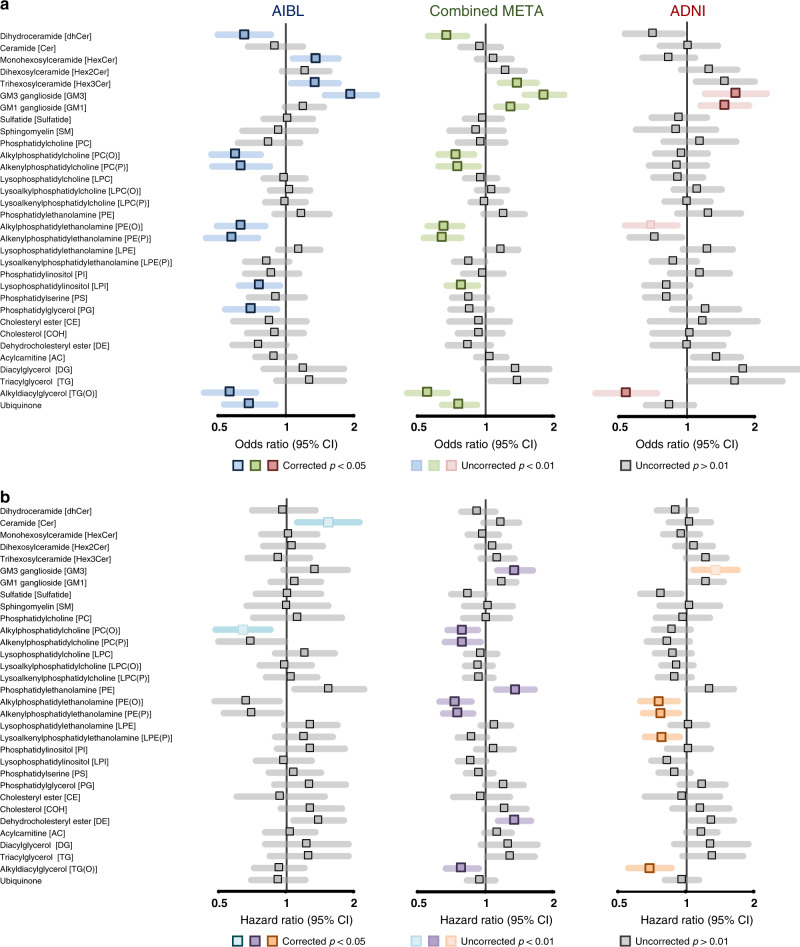
Associations of lipid class totals with prevalent and incident Alzheimer’s disease. Forest plots of lipid class associations for **a** prevalent Alzheimer’s disease (logistic regression, AIBL = 268 cases, 696 control, ADNI = 178 cases, 210 controls) and **b** incident Alzheimer’s disease (Cox regression, AIBL = 68 cases, 714 controls, ADNI = 166 cases, 397 controls). Lipid classes are generated by the sum of each individual species measured in each class. Regressions are adjusted for age, sex, BMI, total cholesterol, HDL-C, triglycerides, number of APOE4 alleles, statin use and omega-3 supplementation. AIBL was further adjusted for time points (only in logistic regression analysis) and site of blood collection. ADNI was further adjusted for fasting status.

**Fig. 3:**
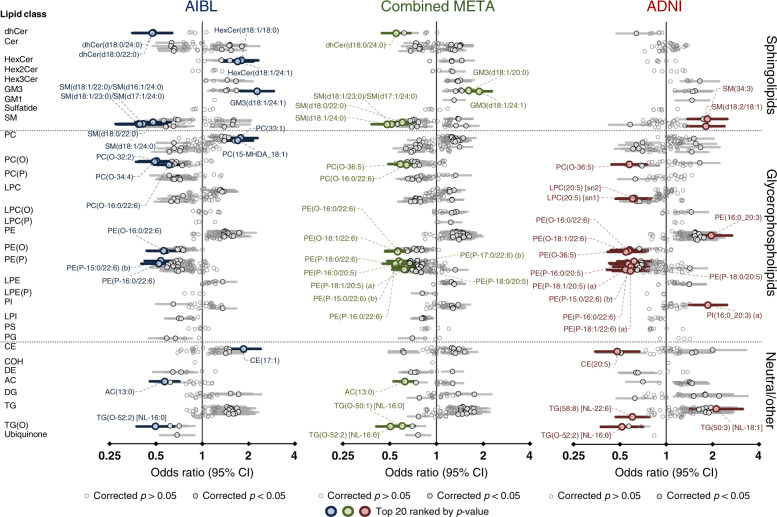
Associations of individual lipid species with prevalent Alzheimer’s disease. Forest plot outlining the logistic regression results of individual species, between controls and prevalent AD in both the AIBL (blue *n* = 268 cases, 696 controls) and ADNI (red, *n* = 178 cases, 210 controls) cohorts with the combined meta-analysis in the middle (green). *P* value was corrected for multiple comparison using approach by Benjamini and Hochberg. Covariates include age, sex, BMI, total cholesterol, HDL-C, triglycerides, number of APOE4 alleles, statin use and omega-3 supplementation. Additional covariates for AIBL include site of blood collection and time point while ADNI includes fasting status. Open circles, not significant; closed dark circles, significant after FDR correction; coloured circles, top 20 associations ranked by *p* value.

After adjustment for covariates (including age, sex, body mass index (BMI), total cholesterol, HDL-C, triglycerides, site of sample collection, APOE ε4 alleles, omega-3 supplementation and statin use). There were 12 and 3 classes significantly associated with AD in the AIBL and ADNI1 cohorts, respectively, after false discovery rate (FDR) correction (Fig. 2), corresponding to 147 and 87 lipids, respectively (219 and 157 uncorrected, Fig. 3). Meta-analysis using a fixed-effects model identified 197 lipids and 11 classes associated with AD between both cohorts (Figs. 2 and 3). The lipid classes associated were predominately from the sphingolipid classes: dihydroceramides (dhCer), trihexosylceramides (Hex3Cer), GM_3_ gangliosides (GM_3_), GM_1_ gangliosides (GM_1_) and ether lipids classes: alkylphosphatidylcholine [PC(O)], alkenylphosphatidylcholine [PC(P)], alkylphosphatidylethanolamine [PE(O)], alkenylphosphatidylethanolamine [PE(P)], alkyldiacylglycerol [TG(O)].

While all plasmalogens (alkenyl classes) are ether lipids, not all ether lipids are plasmalogens. This distinction is important when factoring in their biological significance. Independent of multiple covariates, the majority of the ether lipid classes were negatively associated with AD (Figs. 2 and 3a). This effect is compounded when the ether lipids are esterified with omega-3 fatty acids, such as the 22:6 acyl chain (docosahexaenoic acid, DHA). However, it should be noted that ether lipids with polyunsaturated fatty acids other than omega-3 were still negatively associated with AD (Fig. 3b and Supplementary Table 3), highlighting that the effect is not necessarily driven by omega-3 fatty acids. A non-glycerophospholipid subclass of ether lipids, alkyldiacylglycerols, TG(O), were negatively associated with AD, despite the species measured predominately containing saturated or monounsaturated species (Figs. 2 and 3).

Ceramide and sphingomyelin species presented with both positive and negative associations with AD resulting in no significant associations at the class level. In both cohorts, dhCer was notability negatively associated with AD. Negative associations were primarily driven by 22:0 and 24:0 saturated species in the sphingolipid *n*-acyl moiety, while positive associations were predominately the shorter 18:0, 20:0 and monounsaturated 24:1 species. The sphingoid base had no apparent influence on the association in AD. This sphingolipid pattern in AD is much weaker in the ADNI1 cohort (Fig. 3 and Supplementary Table 3).

This effect was also seen for other complex sphingolipids, where a positive association or trend was observed at the class level (monohexosylceramide, HexCer, dihexosyolceramide, Hex2Cer, Hex3Cer, GM_3_ and G_M1_ gangliosides) in both AIBL and ADNI cohorts (Fig. 2), but individual species within these classes present with the same opposing relationship (negative association with the *n*-acyl chains 22:0 and 24:0, and positive association with the 18:0, 20:0 and 24:1 species). This combined trend resulted in no association with 22:0 and 24:0 *n*-acyl sphingolipids but an increased association in sphingolipids with *n*-acyl chains 18:0, 20:0 and 24:1, such as GM_3_(d18:1/24:1) which has the strongest positive association with AD (Fig. 3 and Supplementary Table 3).

A notable pattern observed in both AIBL and ADNI cohorts, even after adjustment for clinical measures of cholesterol and triglycerides (TGs), was the positive associations of the lipid classes phosphatidylethanolamine (PE) and TG (Fig. 3). This effect has been noted in diseases where dyslipidemia is prevalent such as type 2 diabetes^23^.

### Similar lipids are associated with cross sectional and longitudinal analysis of AD in both cohorts

**Fig. 4:**
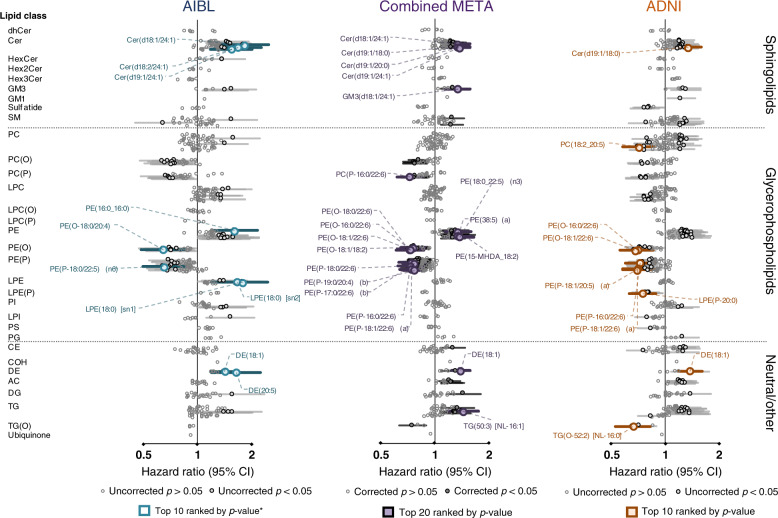
Associations of individual lipid species with future onset Alzheimer’s disease. Forest plot outlining the Cox regression results of individual species, between non-converters and future converters in both the AIBL (cyan *n* = 68 cases, 714 controls) and ADNI (orange, *n* = 166 cases, 397 controls) cohorts with the combined meta-analysis in the middle (purple). *P* value was corrected for multiple comparison using approach by Benjamini and Hochberg. Covariates include age (set as timescale), sex, BMI, total cholesterol, HDL-C, triglycerides, number of APOE4 alleles, statin use and omega-3 supplementation. Open circles, not significant; closed dark circles, uncorrected *p* value; coloured squares, top 10/20 associations ranked by *p* value.

Using Cox regression models, we explored lipids associated with the risk of developing AD in the future. Baseline characteristics of this analysis are presented in Supplementary Table 1. We noted that in both cohorts, there was also a higher proportion of individuals with mild cognitive impairment (MCI) in the conversion group. After adjustment for covariates (age as time scale, sex, BMI, total cholesterol, HDL-C, TGs, site of sample collection, APOE ε4 alleles, omega-3 supplementation and statin use), there were 71 species associated with incident AD in the meta-analysis (Fig. 4 and Supplementary Table 4). One hundred and sixty-one lipids had uncorrected *p* values < 0.05 and the majority of these show the same direction of association as observed for AD (Fig. 4a). These include individual species from the ether lipids, sphingolipids, PE and TG classes. If we further adjust for individuals diagnosed with MCI, only three species remain significant after FDR correction, the sterol ester DE(18:1) and two plasmalogen species.

### Multivariate modelling to identify lipids important in diagnosing prevalent or predicting future onset of AD

Due to the feature selection process (selecting features based on frequency incorporating into a multivariate model), we observed that informatively prognostic lipids, but are otherwise highly correlated, would frequently be selected once in each model interchangeably, reducing their individual frequency count. We utilised the correlation data to identify lipid clusters of highly correlated species. The frequency of these clusters were then used to rank (sum of individual incorporations), with the most incorporated lipid in each cluster used as the representative for the multivariate models (Supplementary Table 5).

In the disease classification model where AIBL was the discovery set and ADNI the replication, we observed a final concordance statistic (*C*-statistic) of 0.752 (0.747–0.757) through the incorporation of 10 lipid species on top of the base model of age, sex, BMI and *APOE* ε4 count (*C*-statistic of 0.731 [0.726–0.736]) with a Net Reclassification Index (NRI) of 0.40 when tested on the ADNI study. For incident AD, we observed a final *C*-statistic of 0.675 (0.671–0.680), up from 0.644 (0.640–0.648) obtained from the base model alone (NRI of 0.40). The summary of results is presented in Table 2.

**Table 2:** Summary of modelling statistics.

		Model	*C*-statistic 95% CI	Net Reclassification Index (total)	Net Reclassification Index (event)
Diagnosis of AD	Discovery: AIBL Replication: ADNI	Age + Sex + BMI + APOE	0.731 (0.726–0.736)	–	–
		Age + Sex + BMI + APOE + 10 lipids^a^	0.752 (0.747–0.757)	0.40 (0.38 – 0.42)	0.19 (0.17–0.20)
	Discovery: ADNI Replication: AIBL	Age + Sex + BMI + APOE	0.820 (0.817–0.823)	–	–
		Age + Sex + BMI + APOE + 10 lipids^b^	0.869 (0.866–0.871)	0.84 (0.82–0.85)	0.44 (0.42–0.45)
Future onset of AD	Discovery: AIBL Replication: ADNI	Age + Sex + BMI + APOE	0.644 (0.640–0.648)	–	–
		Age + Sex + BMI + APOE + 5 lipids^c^	0.675 (0.671–0.680)	0.25 (0.23–0.27)	0.05 (0.04–0.07)
	Discovery: ADNI Replication: AIBL	Age + Sex + BMI + APOE	0.716 (0.709–0.723)	–	–
		Age + Sex + BMI + APOE + 5 lipids^d^	0.733 (0.727–0.740)	0.45 (0.41–0.48)	0.17 (0.14–0.21)

^a^Lipid model comprises GM3(d18:1/24:1), PC(O-32:2), PC(15-MHDA_18:1), AC(13:0), PC(P-18:0/22:5), CE(17:0), PC(O-38:5), Cer(d18:2/26:0), PC(P-16:0/18:2) and PC(P-16:0/20:4).

^b^Lipid model comprises PE(O-36:5), SM(d18:2/18:1), PC(O-40:7) (b), SM(43:1), PE(16:0_18:3) (b), CE(24:5), LPC(20:2) [sn1], PS(38:4), GM3(d18:1/20:0) and GM3(d18:1/24:0).

^c^Lipid model comprises DE(18:1), Cer(d18:1/24:1), LPE(18:0) [sn1], Cer(d19:1/24:1) and SM(41:0).

^d^Lipid model comprises DE(18:1), TG(O-52:2) [NL-16:0], PE(16:0_20:3), Cer(d19:1/18:0) and Hex3Cer(d18:1/22:0).

In the parallel analysis, where the ADNI was the discovery and AIBL was the replication, the disease classification model had a final *C*-statistic of 0.869 (0.866–0.871) through the incorporation of 10 lipid species on top of the base model of age, sex, BMI and *APOE* ε4 count (*C*-statistic of 0.820 [0.817–0.823]) with an NRI of 0.84 when tested on the AIBL study. For prediction of future disease onset, we observed a final *C*-statistic of 0.733 (0.727–0.740), up from 0.716 (0.709–0.723) obtained from the base model alone (NRI of 0.45). The summary of results is presented in Table 2.

## Discussion

Here we present our initial results from the lipidomic analysis of the AIBL and ADNI cohorts. Our goal was to define the complex associations between lipids and AD using our updated methodology by examining each cohort independently and subsequently combining them in a single meta-analysis. The independent nature of these two studies allows for confidence in identifying lipids that are important in AD pathology.

The positive associations of PE, diacylglycerol and triacylglycerol with AD is similar to the associations seen with both pre-diabetes and type 2 diabetes^23^. These species tend to correlate closely with clinically elevated levels of TGs and reduced HDL cholesterol (dyslipidemia). Adjustment for these clinical lipid measures resulted in a loss of significance at the class level; however, multiple species within these classes retained a significant association with AD (Fig. 3). Interestingly, we observe mostly monounsaturated acyl species driving the associations within these classes. Several studies have highlighted the association of dyslipidemia and insulin resistance with the risk of both vascular dementia and AD^24,25^. It may also be possible that the positive association observed with the PE class is a compensatory increase due to the decreases of PE(P) species, a phenomenon that has been reported in plasmalogen-deficient mice^26^.

A key difference between plasmalogens and non-plasmalogen ether lipids is the vinyl-ether bond in the *sn1* position, which is susceptible to oxidation^27^. Little of the recently reported metabolomic literature with respect to AD has differentiated these two classes^5,14–17^. To further complicate this issue, studies which have obtained data from unit resolution mass spectrometers were unable to differentiate these species from isobaric species (odd numbered di-acyl lipids), unless extensive chromatography has also been employed. For example, PC(35:2) has a mass difference of 0.036 Da from PC(O-36:2) and PC(P-36:1), of which the latter two share identical masses (isomeric). When measured on a unit-resolution instrument without chromatography^5^, these all contribute to the same signal. This can raise issues in instances where these species associate differently with the outcome. This is exemplified in the inverse relationship of odd-chain and ether lipids with AD (Supplementary Table 3), highlighting the importance of differentiating these species.

It has been proposed that plasmalogens act as endogenous antioxidants through their vinyl-ether bond and that increased oxidative stress may explain the lower levels observed in AD patients^28^. However, both plasmalogen and non-plasmalogen ether species showed similar associations with AD. Alkyl ether lipids are less susceptible to oxidation compared to their plasmenyl counterparts, suggesting associations with AD more likely reflect changes to the biosynthetic pathway. In support of this, Grimm et al.^29^ reported dysregulation of the peroxisomal enzymes relating to plasmalogen synthesis in AD. Plasmalogens have been reported to be involved in several physiological functions including maintenance of lipid raft domains^30^ which are important for secretase function, responsible for Aβ production^31^, cholesterol efflux^32^ and cellular survival^33^. Importantly, peripheral intravenous administration of plasmalogens has been shown to inhibit Aβ accumulation in a study involving neuroinflammation^34^. Impairment to the synthetic pathway of ether lipids may have deleterious downstream effects, and, in fact, these species have been proposed as potential therapeutic compounds^35^.

We have observed diverse associations of specific *n*-acylated ceramides with AD: positive associations were observed with species containing 18:0, 20:0 and 24:1 fatty acids, whereas negative or neutral associations were observed for species containing 22:0, 24:0 and 26:0 fatty acids, irrespective of the sphingoid base. In contrast, significant associations of sphingolipids with incident AD were only observed for species containing nervonic acid (24:1). This effect was much stronger in the AIBL study, while ADNI exhibited similar trends but were not significant after FDR correction. Despite this, the meta-analysis of sphingolipid associations resulted in much more power than utilising the AIBL study alone (Supplementary Table 3).

Synthesis of 24:1 is likely through elongation of 18:1 which itself is synthesised through unsaturation via stearoyl-CoA desaturase 1 (SCD-1). Interestingly, increased SCD-1 activity has previously been associated with AD^36^, and this may be contributing to the increased abundance of these monounsaturated lipid species. We observed similar positive associations with 18:1 relative to 18:0 in many of our measured lipid species, particularly species with a single fatty acid (e.g. LPC and CE species, Supplementary Table 3). Interestingly SCD-1 is associated with insulin resistance and adiposity, and mouse experiments have shown that disrupting SCD-1 function can potentially reduce body adiposity and improve insulin sensitivity^37^. This is relevant as obesity, insulin resistance and diabetes type II are all likely linked to increased risk of AD^38^

Specific ceramide synthases are responsible for the *n*-acylation of ceramide species, in particular, ceramide synthase 2 (20:0 to 26:0) and 3 (22:0 to 26:0)^39^. Decreases in the activity of ceramide synthase 2 (CerS2) have been observed in AD, early in pathogenesis^40^. Thus, the negative association of Cer(22:0), Cer(24:0) and Cer(26:0) may be driven by decreases of CerS2, while the positive association of Cer(24:1) driven by SCD-1 cancels out the negative association observed with CerS2, which would, on its own, be expected to result in decreased levels of this species.

Gangliosides are a group of sphingolipids with oligosaccharide groups linked to the sphingoid base. GM_3_ gangliosides, the most abundant circulating ganglioside class, is positively associated with AD. Gangliosides have been reported to accelerate Aβ aggregation, leading to deposition in the brain^41^. While the mechanism leading to increased circulating gangliosides is currently not known, gangliosides are not commonly measured, and thus far no other reports have described an association between circulating GM_3_ gangliosides and AD.

The demographic and study design differences between the AIBL and ADNI cohorts presented a unique opportunity to validate some of the lipid markers in this study. Despite the geological differences with the two studies, the cross-sectional associations identify fairly similar associations and patterns within the lipidome and disease outcome. However, one challenge was the study design differences, where the distribution of control, MCI and AD cases were quite different (approximately 7:1:2.5 for AIBL and 1:2:1 for ADNI) which resulted in some differences of important variables in the multivariate modelling, thus requiring retraining of the coefficients in the replication set despite normalisation. Nonetheless, we were able to identify several lipids that appear to be key in identifying individuals who have, or are at, risk of developing AD. These lipids highlight unique features that provide information on top of easily obtained anthropometric (age, sex, BMI) and biochemical features (*APOE* ε4 alleles). While we were able to test and validate these models across the two cohorts, ultimately a population study will be required to fully assess model performance.

While our lipidomic methodology offers a broad coverage of the lipidome, in the absence of stable isotope standards for each of the 569 lipid species, lipidomics will not provide exact quantification. We include a single internal standard per lipid class to provide quantitative data, while acknowledging that differential response factors will lead to slight offsets of the calculated concentration relative to their true concentration. However, this does not impact the association analyses performed in this study nor the multivariate modelling to predict prevalent and incident AD.

Cross-sectional studies have some inherent limitations. We observed strong associations between 218 plasma lipid species and AD. However, dysregulation of the lipidome can arise from sources other than the disease pathology (reverse causation), for example, dietary and activity changes arising as AD progresses. To address this, we were also able to perform association studies with incident AD where we identified similar, albeit weaker, associations with the majority of the same lipid species.

The AIBL and ADNI cohorts represent two powerful studies to characterise AD and the combination provides the opportunity to validate findings across studies. However, these cohorts were selected to be high risk for AD and so traditional risk factors, including age and APOE genotype, are stronger predictors than in an unselected population. As a consequence, these cohorts likely underestimate the performance of risk models for incident AD and further studies on balanced population cohorts will be required.

To conclude, we have performed one of the most comprehensive lipidomic analysis of AD to date by utilising two large, independent clinical studies in AD. We have provided a holistic picture of lipid dysregulation associated with both prevalent and incident AD. Our plasma lipid dataset expands the scientific literature by providing greater resolution and allowing fine-granular analysis of the lipidome. Here we have highlighted specific changes to the ether lipid pathway, where plasmalogens are not the only drivers of ether lipid associations. Class-wide and species-specific changes highlight the necessity of a broad and detailed assay to capture these minute differences in the lipidome. We have demonstrated the potential of plasma lipids as the markers to improve assessment of prevalent and incident AD, highlighting the importance of these small molecules in both disease prognostics and understanding the metabolic changes occurring with the disease.

## Methods

### Participants

The AIBL study recruited 1112 individuals over the age of 60 years into a longitudinal study^42^. At baseline, this comprised 768 cognitively normal, 133 with mild cognitive impairment and 211 with AD. Time points for blood/data collection were every 18 months from baseline. Detailed description of the participants was adapted from Ellis et al.^42^. We analysed all available fasted plasma samples (4106) from baseline up to the fifth time point. After filtering for missing values and problematic samples, the breakdown of sample numbers used in statistical analysis is presented in Table 1. In total, there were 1073, 963, 731, 702 and 564 samples at baseline, and follow-up times 1–4, respectively. While there were decreasing number of samples at successive time points, this was not completely driven by attrition, as some were missing owing to low sample material or were unobtainable at the start of the lipidomics study.

The ADNI1 study started in 2004 and recruited about 800 individuals at baseline. The initial goal was to recruit 200 participants with mild AD and 200 controls as well as 400 participants with MCI. Study data analysed here were obtained from the ADNI database, which is freely available online (http://adni.loni.usc.edu/). We utilised serum samples from the ADNI1 baseline cohort and follow-up data from the 6-, 12-, 18-, 24- and 36-month recalls for incident AD analysis.

### Classification of disease state

Classification of MCI and AD in the AIBL cohort has been described extensively in previous publications^42^. Clinical criteria used to determine disease status included Mini Mental State Examination score of less than 28, failure on the Logical Memory test (in accordance with the ADNI criteria), other evidence of possible significant cognitive difficulty on neuropsychological testing, a Clinical Dementia Rating score of 0.5 or greater, a medical history suggestive of the presence of illnesses likely to impair cognitive function, an informant or personal history suggestive of impaired cognitive function, or who were consuming medications or other substances that could affect cognition^42^. A more in-depth description of the ADNI cohort diagnostic criteria is reported elsewhere^43^; briefly, AD dementia diagnosis was established using NINDS‐ADRDA criteria for probable AD. Classification as MCI followed the Petersen et al.^44^ criteria described previously.

### Lipid extraction and liquid chromatography mass spectrometry

Lipids were extracted from 10 μl plasma (AIBL) or serum (ADNI), with the addition of an internal standard mix (Supplementary Table 2), using the single-phase butanol/methanol extraction method^45^. In brief, 10 μl of samples were mixed with 100 μl of 1:1 butanol:methanol containing the internal standards^22^, the samples were vortexed, sonicated on a sonicator bath and centrifuged (13,000 × *g*, 10 min). The supernatants were transferred into glass vials and stored at −80 °C. On average, 486 samples were extracted per day. Prior to mass spectrometry analysis, samples were thawed for 1 h at room temperature, vortexed and sonicated on the sonicator bath for 15 min and left to sit at 25 °C for 2 h prior to analysis.

Analysis of plasma extracts was performed on an Agilent 6490 QQQ mass spectrometer with an Agilent 1290 series HPLC system. Mass spectrometry settings and transitions for each lipid class are shown in Supplementary Table 2.

Mass spectrometer running conditions were gas temperature 150 °C, gas flow rate 17 l/min, nebulizer gas pressure 20 psi, sheath gas temperature 200 °C, capillary voltage 3500 V and sheath gas flow 10 l/min. Isolation widths for Q1 and Q3 were set to “unit” resolution (0.7 amu). The Agilent 1290 HPLC conditions were as follows: The composition of running solvents A and B comprised 50:30:20 and 1:9:90 water, acetonitrile and isopropanol, respectively. A ZORBAX eclipse plus C18 (2.1 × 100 mm 1.8 mm, Agilent) column was used. Solvents were run at a flow rate of 0.4 ml/min with the column compartment temperature set to 60 °C. The solvent gradient was as follows: starting at 10% B and increasing to 45% B over 2.7 min (while diverting to waste for the first minute), then to 53% B over 0.1 min, to 65% B over 6.2 min, to 89% B over 0.1 min, to 92% B over 1.9 min and finally to 100% over 0.1 min. The solvent was then held at 100% B for 0.8 min and returned to 10% B over 0.1 min (a combined total of 12 min). The column was re-equilibrated in 10% B at an adjusted flow rate (0.4 ml/min for 0.9 min, then 0.6 ml/min for 1 min, then back to 0.4 ml/min for 0.9 min) prior to the next injection.

Additional experiments using pooled samples were utilised under varying conditions to get acyl composition data. To obtain further structural detail of isomeric and isobaric lipid species under the presented chromatographic conditions, we performed additional fragmentation experiments using pooled samples^22^. The additional experiments to characterise the lipid species include acid hydrolysis, which selectively depletes plasmalogens leaving non-plasmalogen ether species intact, fragmentation in the presence of lithium ions in positive ionisation mode and fragmentation in negative ionisation mode, both of which yield characteristic product ions for structural elucidation under our reported analytical conditions^22^.

### Data integration, batch alignment and statistical analysis

Peak area of the lipid species was related to the internal standards to generate concentration data. To remove technical batch variation, the lipid data in each analytical batch (approximately 486 samples a batch) were aligned by using the median value of each lipid of the plasma quality control samples. As most lipid species were positively skewed, final lipid concentrations were log_10_ transformed prior to statistical analysis. Lipid class totals were generated by summing the individual species within each class.

All statistical analyses were performed in the R statistical platform (version 3.4.1+). For logistic and Cox regression, lipid data were scaled by the standard deviation and mean centred within each cohort. When examining associations with prevalent disease, we excluded MCI individuals from the analysis in both studies to provide a clean distinction between disease and non-disease. Furthermore, to maximise the number of AD samples in the prevalent disease analysis, we utilised the last acquired sample for each participants and have added covariates corresponding to each time point to adjust for possible confounding effects.

For examining incident disease analysis, we utilised the baseline samples only. This analysis included healthy and MCI individuals. For both analyses, the meta-analysis was performed using the “meta” package in R and was performed using a fixed effects model.

Correlation analysis was performed using Spearman correlation, with hierarchical clustering of co-linear lipids (complete distance method) followed by tree cutting using the Dynamic cut R package (hybrid method), resulting in 150 and 148 clusters for the AIBL and ADNI lipid data, respectively.

Common covariates utilised in statistical analysis include age, sex, BMI, HDL cholesterol, total cholesterol, clinical TGs, statin use and omega-3 supplementation. FDR correction was performed using the method of Benjamini and Hochberg^46^ using the nominal *p* values from each univariate analysis (569 comparisons for individual lipid species and 32 comparisons for lipid classes). Meta-analysis to combine the results between the two cohorts was conducted using an inverse-variance weighted averaged fixed effect meta-analysis, with similar FDR corrections applied to the nominal *p* values. For modelling, each cohort was *Z*-scored independently.

### Multivariate modelling of AD

We leveraged the availability of two independent cohorts to train and test different lipid models to diagnose prevalent disease (logistic models) and identify individuals at risk of future AD onset (Cox models). We used one cohort to define a set of features (discovery) for a multivariate model (common clinical variables + multiple lipid species) and used the second cohort to test these features for their predictive performance (replication). We then repeated this process switching the cohorts around.

With the AIBL cohort, we used a 2:1 age–sex-matched baseline subset (Supplementary Table 1) due to the low incidence of conversion and the large age difference between converters and non-converters within the cohort. Similarly, we did not adjust for MCI in these models to better allow the predictive performance of the lipidomic markers to be assessed. Due to the different population distribution and characteristics of the two cohorts (AIBL and ADNI) we retrained the model (using the selected features) on the replication cohort. This was also done in a cross-validated framework to avoid overfitting.

To define a feature list in the discovery cohort, we generated a series of models using forward stepwise regression, adding lipid species to the existing covariates (to a maximum of 5 lipids for the Cox model and 10 for the logistic model), while minimising the Akaike Information Criterion, within a 10-fold cross validation framework.

We rationalised that a stepwise approach would help us to maximise model performance while incorporating a minimum number of lipid species into the model, producing a simpler model to minimise the potential of overfitting. We used the frequency of incorporation into the training models, after accounting for clustering of co-linear lipids, to define a set of features that were subsequently validated in the replication cohort.

The need to consider highly correlated lipids within clusters in our selection process was due to the tendency of these lipid species to be selected in different cross-validation folds, and thus reduce the ranking of these correlated lipid species despite being strong predictors of AD. By considering these correlated lipids within clusters, ranking the clusters and selecting only the top ranked lipid within each cluster, we optimised the ranking of the most powerful predictors while avoiding the selection of highly correlated lipid species.

For replication, the features selected from the discovery cohort were modelled in the replication cohort, also within a 10-fold cross-validation framework. The *C*-statistic and NRI were calculated after 200 repeats from the replication results. This process was repeated such that AIBL and ADNI were utilised as both discovery and replication sets.

### Naming convention of lipid species

The lipid naming convention used here follows the guidelines established by the Lipid Maps Consortium and the shorthand notation established by Liebisch et al.^47–49^. We identified several species of interest that are not structurally resolved. These species separated chromatographically but incompletely characterised were labelled with an (a) or (b) to differentiate them, for example PC(P-17:0/20:4) (a) and PC(P-17:0/20:4) (b) where (a) and (b) represent the elution order. Separated isoforms that contain a 16:0 methyl branched fatty acid are presented as MHDA (methylhexadecanoic acid).

### Reporting summary

Further information on research design is available in the Nature Research Reporting Summary linked to this article.

## Supplementary information

Supplementary Table 1 - 6

Reporting Summary

## Data Availability

Data to support these findings are available online and upon request. The entire ADNI lipidomic and clinical characteristic data are available online (adni.loni.usc.edu, specifically, ADMC Lipidomics Meikle Lab Baseline Data Matrix [ADNI1]) and the remaining data (AIBL) used in this study are available from both the corresponding authors and online at https://aibl.csiro.au upon reasonable request. Source data are provided with this paper.

## Source data

Source Data File
