# Supplementary material for: Concordant peripheral lipidome signatures in two large clinical studies of Alzheimer’s disease
Source: Nat Commun. 2020 Nov 10;11:5698. doi: 10.1038/s41467-020-19473-7 (PMC7655942; doi:10.1038/s41467-020-19473-7)
Supplement: Supplementary file 2 — Reporting Summary [file 41467_2020_19473_MOESM2_ESM.pdf]

nature research

Corresponding author(s): Peter J Meikle
Ralph N Martins

Last updated by author(s): 12/10/2020

Reporting Summary

Nature Research wishes to improve the reproducibility of the work that we publish. This form provides structure for consistency and transparency in reporting. For further information on Nature Research policies, see Authors & Referees and the Editorial Policy Checklist.

Statistics

For all statistical analyses, confirm that the following items are present in the figure legend, table legend, main text, or Methods section.

n/a	Confirmed	
☐	☒	The exact sample size (*n*) for each experimental group/condition, given as a discrete number and unit of measurement
☐	☒	A statement on whether measurements were taken from distinct samples or whether the same sample was measured repeatedly
☐	☒	The statistical test(s) used AND whether they are one- or two-sided<br>*Only common tests should be described solely by name; describe more complex techniques in the Methods section.*
☐	☒	A description of all covariates tested
☐	☒	A description of any assumptions or corrections, such as tests of normality and adjustment for multiple comparisons
☐	☒	A full description of the statistical parameters including central tendency (e.g. means) or other basic estimates (e.g. regression coefficient) AND variation (e.g. standard deviation) or associated estimates of uncertainty (e.g. confidence intervals)
☐	☒	For null hypothesis testing, the test statistic (e.g. F, t, r) with confidence intervals, effect sizes, degrees of freedom and P value noted<br>*Give P values as exact values whenever suitable.*
☒	☐	For Bayesian analysis, information on the choice of priors and Markov chain Monte Carlo settings
☒	☐	For hierarchical and complex designs, identification of the appropriate level for tests and full reporting of outcomes
☐	☒	Estimates of effect sizes (e.g. Cohen's *d*, Pearson's *r*), indicating how they were calculated

Our web collection on statistics for biologists contains articles on many of the points above.

Software and code

Policy information about availability of computer code

Data collection	No software was used
Data analysis	Data anaysis was performed on open-source software (R version 3.4.1 and above)

For manuscripts utilizing custom algorithms or software that are central to the research but not yet described in published literature, software must be made available to editors/reviewers. We strongly encourage code deposition in a community repository (e.g. GitHub). See the Nature Research guidelines for submitting code & software for further information.

Data

Policy information about availability of data

All manuscripts must include a data availability statement. This statement should provide the following information, where applicable:

- Accession codes, unique identifiers, or web links for publicly available datasets
- A list of figures that have associated raw data
- A description of any restrictions on data availability

Data to support this finding is available online and upon request. The entire ADNI lipidomic and clinical characteristic data is available online (adni.loni.usc.edu) and the remaining data (ABIL) used in this study is available from both the corresponding authors and online at https://aibl.csiro.au upon reasonable request. This has been added to the manuscript.

Field-specific reporting

Please select the one below that is the best fit for your research. If you are not sure, read the appropriate sections before making your selection.

☒ Life sciences ☐ Behavioural & social sciences ☐ Ecological, evolutionary & environmental sciences

For a reference copy of the document with all sections, see nature.com/documents/nr-reporting-summary-flat.pdf

Life sciences study design

All studies must disclose on these points even when the disclosure is negative.

Sample size	Sample size was obtained based on the availability of samples for the study studies described here (AIBL and ADNI). Sufficient size was determined prior to analysis, where prior experience indicated the power of the sample size .
Data exclusions	We obtained all available samples from the AIBL and ADNI study neccesariy for this study. Any samples that did not have sufficient plasma/ serum for lipidomic analysis was excluded from the analysis. Samples that had missing data necessarily for the analysis and which were unable to be imputed was excluded from the analysis. The final number of samples used is described in the demographic table and
Replication	We replicated this analysis using two independent international cohorts. Analysis was done both on the AIBL and ADNI cohorts independently, with the results compared using a fixed effect meta analysis. This replication was performed for all statistical analysis relating to disease outcomes in this study. Modeling done on either cohort was tested in the other with both results presented.
Randomization	Samples were randomized prior to lipidomic analysis. For both the AIBL and ADNI studies, randomisation was not required as group assignment was based off diagnosis of disease (via clinical review panels, ABIL described in Ellis et al. 2009 and ADNI described in Weiner et al. 2010). Confounding variables (such as differences in age between healthy and diseased individuals) were controlled for in all regression and modeling analysis as covariates.
Blinding	Samples from both cohorts (AIBL and ADNI) were blinded to the authors who were involved in lipidomic analysis, up until the statistical analysis point. Similar to randomization, group assignments were not blinded as disease status was used as the assignment variable.

Reporting for specific materials, systems and methods

We require information from authors about some types of materials, experimental systems and methods used in many studies. Here, indicate whether each material, system or method listed is relevant to your study. If you are not sure if a list item applies to your research, read the appropriate section before selecting a response.

Materials & experimental systems

n/a	Involved in the study	
☒	☐	Antibodies
☒	☐	Eukaryotic cell lines
☒	☐	Palaeontology
☒	☐	Animals and other organisms
☐	☒	Human research participants
☒	☐	Clinical data

Methods

n/a	Involved in the study	
☒	☐	ChIP-seq
☒	☐	Flow cytometry
☒	☐	MRI-based neuroimaging

Human research participants

Policy information about studies involving human research participants

Population characteristics	The two main cohort used in this paper, ADNI and AIBL, are derived from an elderly based population (age > 50). AIBL was recruited within Australia (predominately Melbourne and Perth) whereas ADNI was recruited in the United States. Detailed clinical variables (important to this study) are detailed in the manuscript (Table 1 and Supplementary Table 1) and also their respective published papers (AIBL, Ellis et al 2009 and ADNI, Weiner et al. 2010)
Recruitment	Our study itself does not directly involve participant recruitment or design. The recruitment for this study is extensively described in their relative published papers (AIBL, Ellis et al 2009 and ADNI, Weiner et al. 2010). The recruitment strategies employed by each study is unlikely to have any impact on this study and its findings.
Ethics oversight	Our study itself does not directly involve the participants but samples stored from their recruitment. For the lipidomics analysis presented in this study, the ABIL study was deemed low risk (The Alfred Ethics Committee, Project 183/19) while ADNI was deemed RESEARCH NOT INVOLVING HUMAN SUBJECTS (Duke Institute review board, ID: Pro00053208).

Note that full information on the approval of the study protocol must also be provided in the manuscript.
